# Metachronous double primary malignant tumors with nasopharyngeal carcinoma and diffuse malignant peritoneal mesothelioma accompanied with paraneoplastic syndromes treated with nivolumab: A case report

**DOI:** 10.1097/MD.0000000000034349

**Published:** 2023-07-28

**Authors:** Liang-Ke Tang, Zhi-Ke Li, Ya-Lang Xiang, Dai-Yuan Ma, Guo-Bo Du

**Affiliations:** a Department of Oncology, Affiliated Hospital of North Sichuan Medical College, Nanchong, China.

**Keywords:** malignant peritoneal mesothelioma, metachronous double primary malignant tumors, NPC, paraneoplastic syndromes, PD-1

## Abstract

**Rationale:**

Multiple primary malignant tumors are rare and challenging to diagnose. Diffuse malignant peritoneal mesothelioma (DMPM) originate from the peritoneum, which lacks specific clinical manifestations and is difficult to diagnose, with a short survival about 10 to 13 months for inoperable ones. This is the first report of metachronous double primary malignant tumors in nasopharyngeal carcinoma and DMPM accompanied with paraneoplastic syndromes.

**Patient concerns:**

A 61-year-old man presented with abdominal discomfort with a history of nasopharyngeal carcinoma 5 years ago.

**Diagnoses:**

The diagnosis of DMPM was finally confirmed by laparoscopic mesenteric biopsies. Paraneoplastic syndromes including increased platelets were present when diagnosis, followed by increased neutrophils after disease progression.

**Interventions:**

Due to intolerable for surgery, he was treated with pemetrexed combined with nivolumab, intraperitoneal infusion of nivolumab, radiotherapy, anlotinib and maintenance treatment of nivolumab.

**Outcomes:**

Progression-free survival in first line is 12 months, overall survival is 23 months.

**Lessons:**

This indicate that comprehensive treatment including immunotherapy may be helpful for inoperable DMPM patients with nasopharyngeal carcinoma accompanied with paraneoplastic syndromes.

## 1. Introduction

Multiple primary malignant tumors (MPMTs) were first described in 1932.^[1]^ The reported definition of MPMTs is as follows: each neoplasm should be histologically confirmed as malignant; each neoplasm should have unique pathomorphological features; the neoplasms should develop in different places and are disconnected; the suspicion of metastasis must be excluded. The incidence of MPMTs is about 2.4%.^[2]^ Most reports divide MPMTs into synchronous and metachronous with a 6-month’ cutoff.

Nasopharyngeal carcinoma (NPC) is a common cancer associated with the Epstein-Barr virus, but to our best knowledge, there’s no report about MPMTs with NPC and diffuse malignant peritoneal mesothelioma (DMPM). Malignant mesothelioma is a rare tumor mainly arising from plural, peritoneal, and pericardial mesothelium. DMPM accounts for approximately 7% to 20% of all malignant mesotheliomas, with an incidence of about (1–2)/1,000,000.^[3]^ DMPM lacks a specific clinical presentation, abdominal pain, abdominal distension, and wasting are the most common symptoms.^[4]^ The diagnosis of DMPM is challenging, positive in calretinin, CK5/6, D2-40 and CK7 showed by immunohistochemistry is helpful for its diagnosis. The treatment of DMPM is mainly referred to malignant pleural mesothelioma, which include surgery, pemetrexed plus platinum, and intraperitoneal thermal perfusion. Surgery, even cytoreductive surgery, is important for prolonging overall survival (OS). Immunotherapy, such as nivolumab (an antibody against PD-1) has shown efficacy in malignant mesotheliomas, but there’s no report about intraperitoneal infusion with nivolumab in DMPM accompanied with NPC.

Paraneoplastic syndromes aren’t caused by tumor directly, but by cytokines, peptides, hormones secreted, and so on.^[5]^ A high prevalence of thrombocytosis in malignant mesothelioma has been reported, however, neutrophil elevation occurs less frequently, but both two can lead to a worse prognosis.^[6]^ There is currently no report about paraneoplastic syndromes happening in metachronous double primary malignant tumors with NPC and DMPM.

To our best knowledge, this is the first report about metachronous double primary malignant tumors with NPC and DMPM, accompanied with paraneoplastic syndromes, who was successfully treated with pemetrexed combined with nivolumab, intraperitoneal infusion of nivolumab, radiotherapy, anlotinib and maintenance treatment of nivolumab, progression-free survival (PFS) in first line is 12 months, OS is 23 months, which is much longer than the reported inoperable ones. This case suggests that MPMTs with rare tumors, such as DMPM, are more difficult to diagnose. For patients with inoperable DMPM, a combination of immunotherapy, chemotherapy and radiotherapy may result in a longer OS, which needs further research.

## 2. Case presentation

**Figure 1.**
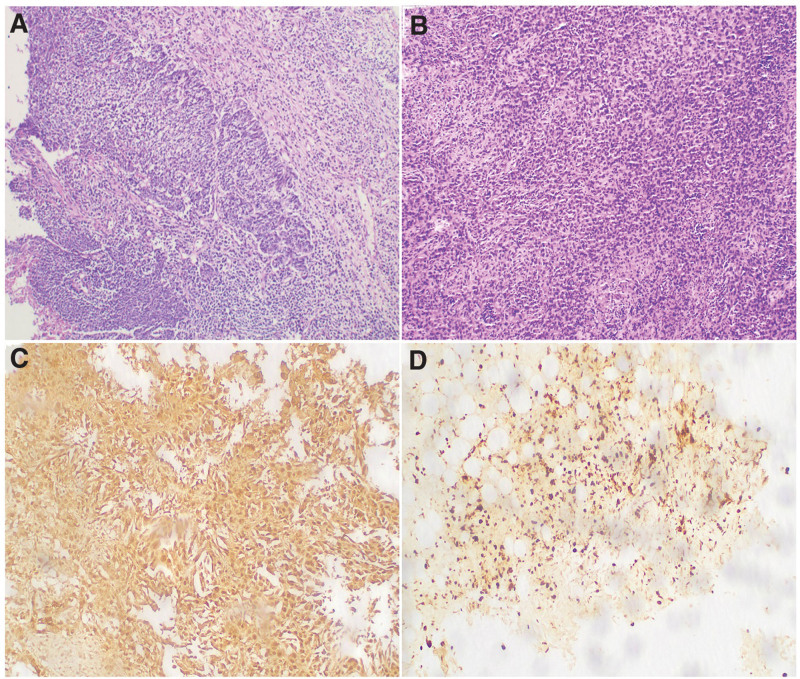
Results of HE staining and immunohistochemistry of the tumors: (A) HE staining of NPC, 100×; (B) HE staining of DMPM, 100×; (C) positive in calretinin of DMPM, ×100; (D) positive in Ki-67 (about 20%), ×100. DMPM = diffuse malignant peritoneal mesothelioma, HE = hematoxylin-eosin, NPC = nasopharyngeal carcinoma.

A 61-year-old man admitted to the hospital due to abdominal distension and pain for more than 1 year in June 2019, he was diagnosed with NPC (Fig. 1A) and treated with radiotherapy and chemotherapy 5 years ago. Abdominal ultrasound and MRIA both suggested that the peritoneum was thickened and the abdominal lymph nodes were enlarged. Positron emission tomography/computed tomography showed no recurrence in the nasopharynx or cervical lymph nodes, but there were multiple suspicious lesions in S5 and S1 segments of the liver, part of the peritoneum, the side of the gastric lesser curvature, and the abdominal lymph nodes. Metastasis from NPC was considered, however, he refused further diagnosis and treatment.

**Figure 2.**
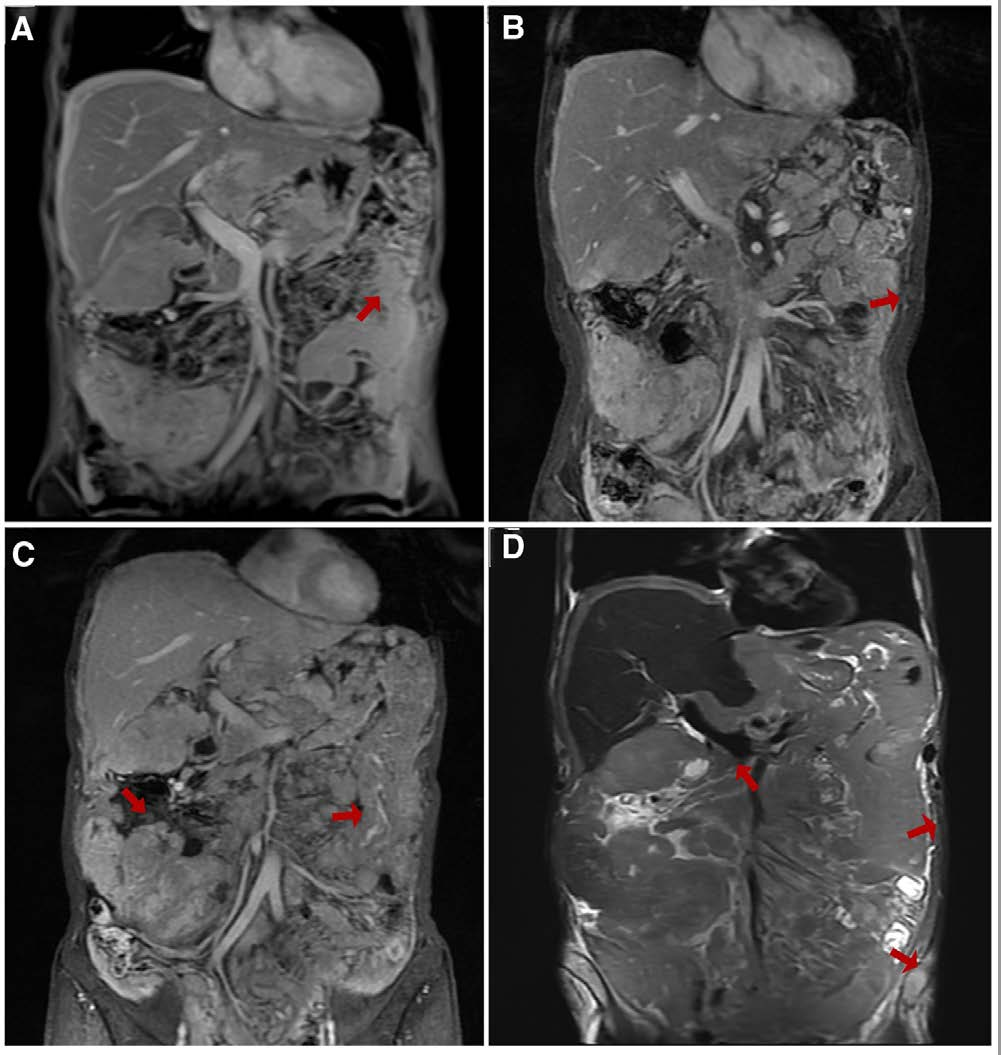
T2 the MRI of DMPM: (A) before pemetrexed plus nivolumab treatment in June 2020; (B) after 6 cycles of pemetrexed combined with nivolumab, the evaluated was SD; (C) after 12 cycles of nivolumab, the evaluated was PD, before radiotherapy; (D) After the combination of anlotinib and nivolumab in June 2020, the evaluated was PD. DMPM = diffuse malignant peritoneal mesothelioma, PD = progressive disease, SD = stable disease.

Then in June 2020, the patient was returned due to aggravation of abdominal distension, and a large amount of ascites were found in his abdomen. One thousand two hundred fifty milliliter of yellowish, slightly turbid fluid was drained by ultrasound-guided puncture, and the amount of fluid drained was more than 1000 mL per day for the following 6 days. Cancer cells were found in the fourth ascites exfoliative cytology, but the source couldn’t be analyzed by immunohistochemistry, due to insufficient cancer cells. The CA-125 in ascites was 116.89 KU/L, platelet was 753 × 10^9^/L, leukocytes was 8.83 × 10^9^/L, and neutrophils was 6.95 × 10^9^/L on June 12, 2020. To reduce the ascites, a anti angiogenic drug (recombinant human endostatin, 60 mg) was given by intraperitoneal infusion. Then a laparoscopic mesenteric biopsy was performed after draining ascites (Fig. 2A). Biopsy results suggested a malignant mesothelioma. Immunohistochemistry showed: calretinin (+), CK5/6 (few, weak +), WT1 (partial +), D2-40 (+), PAX8 (−), CK7 (+), CK20 (−), Ki67 (+, about 20%). According to the results of hematoxylin-eosin staining and immunohistochemistry (Fig. 1B–D), diffuse epithelioid malignant peritoneal mesothelioma was confirmed.

**Figure 3.**
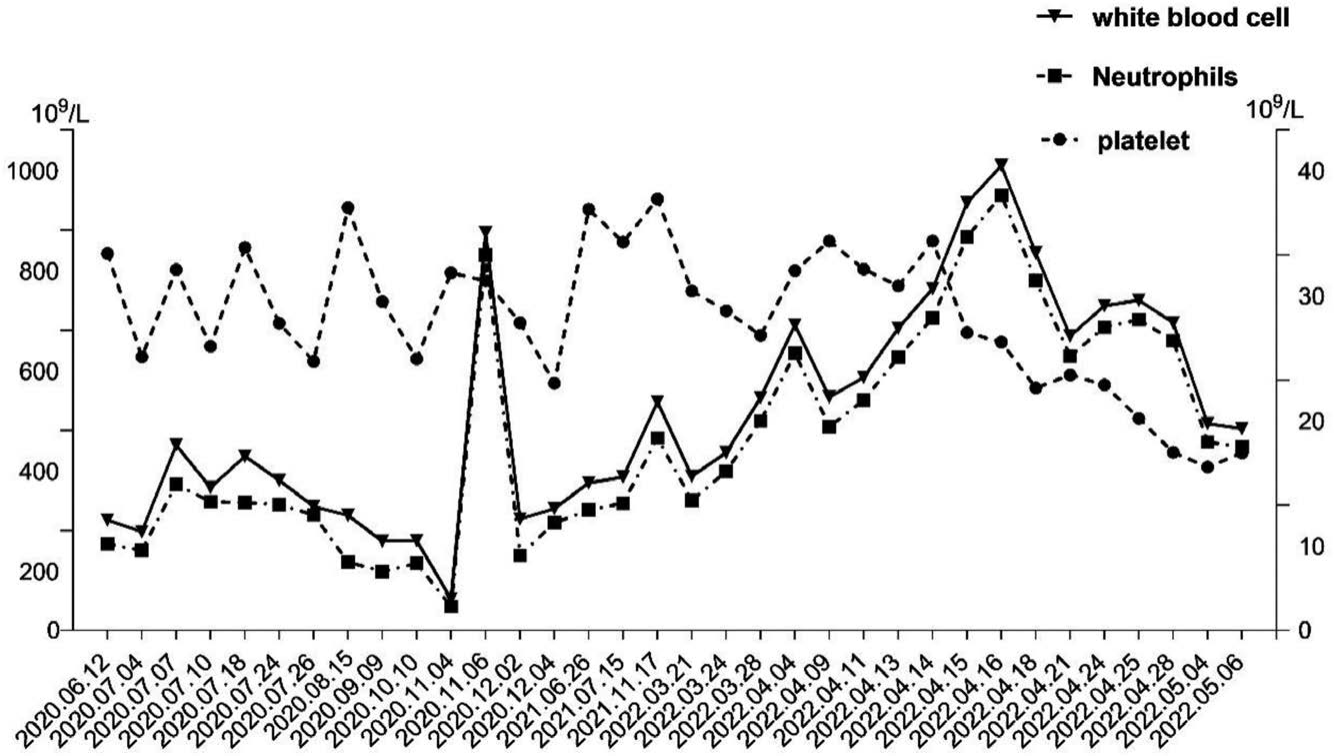
The changes of white blood cells, neutrophils and platelet of the patient from June 2020 to May 2022. The patient underwent leukocyte-increasing treatment due to chemotherapy leukopenia on November 06, 2020.

After the operation, a cycle of chemotherapy (lobaplatin 40 mg plus fluorouracil 1000 mg) was administered intraperitoneally. After an MDT discussion, he was considered inoperable due to poor physical condition, thus the combination of platinum and pemetrexed was suggested. As he refused combination chemotherapy, taking the patient’s opinion into account, he was treated with 6 cycles of pemetrexed (800 mg) plus nivolumab (140 mg) (q3w, from July to December 2020). The best efficacy evaluation was stable disease (SD) (Fig. 2B). The platelets was 494 × 10^9^/L, leukocytes was 9.74 × 10^9^/L, and neutrophils was 8.61 × 10^9^/L on December 24, 2020. As he also refused surgery, so from December 2020 to July 2021, nivolumab maintenance monotherapy was performed every 3 weeks with no progression. But in July 2021, the efficacy evaluation was progressive disease (PD) due to new peritoneal lesions (Fig. 2C), the PFS in first line was 12 months. Palliative radiotherapy (P-GTV:10 Gy/5 Fx) was performed to his large abdominal lesions to enhance the effect of nivolumab. The platelets was 776 × 10^9^/L, leukocytes was 12.27 × 10^9^/L, and neutrophils was 10.18 × 10^9^/L on July 15, 2021. In November 2021, positron emission tomography/computed tomography showed PD of the abdominal lesions, which may have invaded the intestine, so anlotinib (12 mg, qd, d1-14, q3w) was added to nivolumab. At this time, the platelets was 868 × 10^9^/L, leukocytes was 18.28 × 10^9^/L, neutrophils was 15.40 × 10^9^/L. After 3 months, when he felt bloated and the abdominal pain worsened, MRI suggested PD (the lesions of the liver and peritoneum) (Fig. 2D). At this time, the serum C-reactive protein was 195.33 × 10^9^/L, platelets was 678 × 10^9^/L, leukocytes was 12.35 × 10^9^/L and neutrophils was 10.40 × 10^9^/L. He had positive fecal occult blood and the hemoglobin was only 55 g/L, which was considered to be caused by tumor invasion of gastrointestinal tract, thus anlotinib was stopped, but nivolumab was still continued. Then the patient’s peripheral blood cell counts continued to increase, with the highest number of leukocytes was 37.14 × 10^9^/L and neutrophils was 34.80 × 10^9^/L (Fig. 3), interestingly, no infection was detected, and after anti-infection treatment, the neutrophils and leukocytes didn’t drop significantly. Then the patient’s general condition was poor and refused laparotomy or bone aspiration, and he couldn’t tolerate combination therapy. Genetic testing was performed to evaluate whether targeted therapy could be performed, and the results showed that the FGF4 and mTOR were mutated, and the tumor mutational burden was 1.4 mutations/Mb, no targeted therapy was suitable. Then 3 intraperitoneal infusions of nivolumab (20 mg) were administered since April 29, 2022, the localized distension and pain was relieved thereafter, but unfortunately, he passed away on May 26, 2022. The OS was 23 months.

## 3. Discussion

MPMTs is a rare and diagnostically challenging disease, in which the diagnosis of the second tumor often interferes with the first one. NPC is a common tumor whose main cause of failure is distant metastasis. Abdominal metastasis from NPC, which we considered firstly in our case, is about 10% in all NPC metastases, but the peritoneum lesions were finally diagnosed as DMPM. The diagnosis of peritoneal mesothelioma is difficult, with an average time to diagnosis of about 122 days.^[7]^ In this case, the patient refused further examination, resulting in a prolonged diagnosis time of about 360 days. The following may be helpful for the diagnosis: high levels of platelets, IL-6 and CA-125; massive ascites with high levels of IL-6 or CA-125; peritoneal thickening and abdominal lymph nodes showed by MRI; histopathological and immunohistochemical examination results. In the peripheral blood of DMPM patients, CA-125 increased in 53% of patients,^[8]^ while 83% of them had increased platelets. Meanwhile, the ascites puncture is often negative, as only a few malignant cells presenting in ascites, and the diagnosis can only be made from tissue samples ultimately. The pathological subtypes of MPM were classified as epithelioid, biphasic and sarcomatoid subtypes, the epithelial type has the highest incidence, accounting for 50%, with the best prognosis.^[9]^ However, the median survival of inoperable ones is short, only about 10 to 13 months.^[10,11]^ The PFS in first line is 12 months, OS is 23 months in our case, much longer than the reports.

Chemotherapy and hyperthermia intraperitoneal chemotherapy (HIPEC) are the preferred treatment options for inoperable epithelioid peritoneal mesothelioma, which can reduce tumor burden, alleviate clinical symptoms and prolong survival. Main chemotherapy regimen for inoperable DMPM is pemetrexed with or without platinum. Due to the good efficacy in pleural mesothelioma, the combination of nivolumab and ipilimumab is recommended in non-epithelioid peritoneal mesothelioma. Whether the expression of PD-1 and PD-L1 is related to the prognosis of immunotherapy in DMPM is unclear.^[12]^ Some studies have shown that PD-1 is downregulated in DMPM as a result of chemotherapy. This may emphasize the importance of the timing of immunotherapeutic interventions.^[13]^ In our case, the patient accepted nivolumab since diagnosis and had achieved a long PFS and OS. But Immunotherapy is suggested as a subsequent systemic therapy in epithelioid peritoneal mesothelioma. In a study of immunotherapy about advanced DMPM, there was no significant difference in objective response rate and median OS between patients treated with dual-agent and single-agent immunotherapy, both median OS was about 19.1 months. This study included patients with palliative care and cytoreductive surgery+HIPEC, no matter if they had received pemetrexed and platinum or not.^[14]^ At present, platinum is mainly used in HIPEC. Mesothelioma is characterized as a tumor with large infiltration of PD-L1-positive tumor cells and PD-1-positive immune cells. It has been shown that in the presence of anti-PD-L1 antibodies, tumor cells can be targeted by the patient’s own NK cells.^[15]^ This may predict the possibility of the effectiveness of intraperitoneal infusions of nivolumab. Immunotherapy usually ends with PD or intolerable side effects. But studies found that continued use of immunotherapy after PD may lead to tumor reduction or disease stabilization.^[16]^ Therefore, the patient in our case continued to accept nivolumab after PD to prevent accelerated disease progression after discontinuation.

In current reports, the paraneoplastic syndrome in DMPM is mainly thrombocytosis, but in our case, in addition to thrombocytosis, he also underwent increased neutrophils. IL-6 (an inflammatory factor) may be from tumor cells in the peritoneal cavity, so it is often increased locally in the peritoneal cavity without significant changes in the serum. IL-6 is correlated with increased frequency and function of myeloid-derived suppressor cells and can suppress the development of dendritic cells from monocytes.^[17]^ The thrombocytosis in this patient may be related to the stimulation of thrombopoietin by the increased IL-6. What else, neutrophilia may be associated with an increase in granulocyte colony-stimulating factor during disease progression.^[18]^ But unfortunately, we did not check it. Increased platelets, IL-6^[19]^ and neutrophils often indicate a poor prognosis, but our case achieved a long OS, we guess this may owe to early intervention of immunotherapy, which needs further research.

## 4. Conclusion

In conclusion, we firstly report a rare case of MPMTs with NPC and DMPM accompanied with paraneoplastic syndromes, who was successfully treated with pemetrexed plus nivolumab, intraperitoneal infusion of nivolumab, radiotherapy, anlotinib and maintenance treatment of nivolumab. This indicated comprehensive treatment including immunotherapy may be a hopeful choice for inoperable DMPM patients with NPC accompanied with paraneoplastic syndromes, which needs further research.

## Author contributions

**Resources:** Dai-Yuan Ma.

**Supervision:** Dai-Yuan Ma.

**Writing – original draft:** Liang-Ke Tang, Ya-Lang Xiang.

**Writing – review & editing:** Zhi-Ke Li, Guobo Du.
